# Photoacoustic Detection of H_2_ and NH_3_ Using Plasmonic Signal Enhancement in GaN Microcantilevers

**DOI:** 10.3390/mi11070680

**Published:** 2020-07-13

**Authors:** Digangana Khan, Hongmei Li, Ferhat Bayram, Durga Gajula, Goutam Koley

**Affiliations:** 1 Holcombe Department of Electrical and Computer Engineering, Clemson University, Clemson, SC 29634, USA; hongmel@g.clemson.edu (H.L.); fbayram@g.clemson.edu (F.B.); gkoley@clemson.edu (G.K.); 2 School of Electrical and Computer Engineering, Georgia Institute of Technology, Atlanta, GA 30332, USA; gdraophy@gmail.com

**Keywords:** photoacoustics, piezotransistive, plasmonics, gas sensing, Pd nanoparticles, Pt nanoparticles

## Abstract

Photoacoustic (PA) detection of H_2_ and NH_3_ using plasmonic excitation in Pt- and Pd-decorated GaN piezotransistive microcantilevers were investigated using pulsed 520-nm laser illumination. The sensing performances of 1-nm Pt and Pd nanoparticle (NP) deposited cantilever devices were compared, of which the Pd-coated sensor devices exhibited consistently better sensing performance, with lower limit of detection and superior signal-to-noise ratio (SNR) values, compared to the Pt-coated devices. Among the two functionalization layers, Pd-coated devices were found to respond only to H_2_ exposure and not to NH_3_, while Pt-coated devices exhibited repeatable response to both H_2_ and NH_3_ exposures, highlighting the potential of the former in performing selective detection between these reducing gases. Optimization of the device-biasing conditions were found to enhance the detection sensitivity of the sensors.

## 1. Introduction

There is a universal and perpetual need for more efficient and accurate sensors for toxic and flammable gases such as H_2_ and NH_3_. H_2_ is a highly flammable gas that forms an explosive mixture with air at 4.65 vol.% [1], while NH_3_ is a highly toxic gas that has wide usage as a fertilizer and in the manufacturing of plastics and textiles [2]. Hence, constant monitoring of H_2_ and NH_3_ (which can sometimes be produced and present at the same time) with high sensitivity is of extreme importance [1,3,4]. The most common sensing mechanism for such gases is functionalized chemi-resistors [5] or Schottky diodes [6,7]. Metals like Pt and Pd are widely used as the functionalization layer or as the Schottky metal because of their unique capability of adsorbing H_2_ [8,9,10] on the surface, directly, or after dissociating NH_3_ [11,12,13]. This process modifies the surface work function of the metal substrate or the Schottky contact, which can be measured in terms of change in resistivity in chemi-resistors or as a change in the Schottky barrier in chemi-diodes. However, these sensors suffer from several limitations, e.g., thermal instability of the Schottky contacts [14] and degradation of the contact due to exposure to chemicals and poisoning [15]. 

Microelectromechanical system (MEMS) and nanoelectromechanical system (NEMS) devices have been extensively used as physical sensors for measurement of various physical parameters including acceleration, mass, pressure, temperature, strain, and radiation [16,17,18,19,20,21,22]. They have also been utilized in recent years for detection of chemical and biological analytes utilizing functionalized surfaces [23]. The detection methods with functionalized surfaces typically involve physisorption or chemisorption of analyte molecules at the surface causing simple dipolar work function change (physisorption) or charge exchange through bond formation (chemisorption). Both the sorption processes can change the surface work function which can be utilized for highly sensitive detection of gases. Nomani et al. demonstrated the detection of ultra-low concentration of NO_2_ (down to 20 ppb) utilizing the surface work function change in In_2_O_3_ thin films [24], while Singh et al. demonstrated NH_3_ and NO_2_ sensing using graphene/Si chemi-diode with tunable sensitivity [25,26]. Hydrogen detection using Pd- and Pt-functionalized graphene/Si chemi-diode was also demonstrated by Uddin et al. [27]. Microcantilevers, resembling tiny diving boards, have also been extensively utilized for analyte detection with ultra-high sensitivity, taking advantage of their resonant quality factors [28,29]. In 2003, Pinnaduwage et al. reported trace-level explosive detection with Si microcantilevers utilizing the change in surface stress due to surface adsorption [30], while in 2005, Huang et al. showed H_2_ sensing utilizing Pd-alloy cantilever beams by tracing the frequency shift due to H_2_ adsorption [31]. Detection of H_2_ using noncontact surface work function-based measurements (where the surface was functionalized instead of the cantilever) was also reported by Laksmanan et al. [32].

The use of piezotransistive microcantilevers utilizing AlGaN/GaN heterostructures has attracted significant research interest in recent years because of their superior sensitivity in detecting toxic gases and volatile compounds and for several other advantages like their applications in harsh environment and power electronics [33,34,35]. Moreover, AlGaN/GaN heterojunction-based field effect transistors (HFETs), when embedded at the base of a GaN microcantilever, can be used as displacement transducers with a much higher (10–100 times) gauge factor (GF) than the best reported values (~100) [36,37] in their Si counterparts utilizing their unique piezoelectric properties, which are then implemented in highly sensitive photoacoustic (PA) detection of analytes [38,39,40,41,42]. In 2015, Talukdar et al. demonstrated femtoscale level of deflection transduction in ultra-high sensitive PA detection of explosives utilizing AlGaN/GaN heterojunction field effect transistor integrated GaN microcantilevers [42]. 

Plasmonic nanoparticles (NPs) have been used to enhance absorption in opto-electronic devices to amplify their performance [43,44,45]. Recently, we reported on the usage of plasmonic NPs to enhance the sensor signal up to two orders of magnitude in GaN micocantilevers [46]. These NPs, besides enhancing the sensor performance, can also act as the functionalization layer in sensing analytes such as H_2_ [47] or NH_3_ [11,12,13] and have been extensively utilized in surface plasmon resonance (SPR)-based sensors. These sensors offer the unique advantage of noncontact sensing enabling high temperature or harsh environment detection of analytes [48,49], which is a distinct limitation of chemi-resistors or chemi-diodes. However, SPR-based sensors monitor the change in the optical properties of the NPs due to the analyte adsorption that involves cumbersome instruments like polarizers or spectral analyzers. By using the plasmonic nanolayer on GaN microcantilevers, the change in the plasmonic excitation modifies the strength of the PA wave generated, which is then measured in terms of an electrical signal at the HFET output, providing a distinct advantage over the traditional SPR sensing methods, with a much simpler readout technique. 

In this work, we investigated the PA detection of H_2_ and NH_3_ using the variation in plasmonic excitation of Pt and Pd NPs utilizing GaN microcantilevers. A comparison of H_2_ sensing performance between Pt and Pd NPs-coated devices was presented in terms of response time, signal-to-noise ratio (SNR), and limit of detection (LOD). It has been shown that the biasing condition of the HFET can further enhance the LOD in these sensors. It was also demonstrated that, at room temperature, Pd-coated devices exhibit a unique selectivity to H_2_ over NH_3_, while the Pt-coated devices respond to both H_2_ and NH_3_.

## 2. Experimental Details

**Figure 1:**
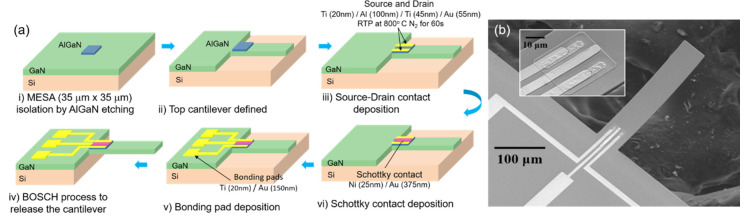
(**a**) Schematic diagram showing the fabrication process flow for AlGaN/GaN heterojunction-based field effect transistor (HFET) integrated piezotransistive GaN microcantilevers. (**b**) SEM image of a fully fabricated GaN cantilever. Scale bar is 100 µm. Inset shows a magnified SEM picture of the HFET embedded at the base of the cantilever. Scale bar is 10 µm.

Microcantilevers were fabricated using III-V nitride epitaxial layers on Si (111) wafer purchased commercially form the NTT Advanced Technology Corporation. The layer structure of the wafer consisted of i-GaN (2 nm)/AlGaN (20 nm, 25% Al)/i-GaN (1 μm)/transition layer (0.3 μm)/Si (111) substrate (675 μm). The schematic flow of the six photolithographic fabrication steps are shown in Figure 1a. In the first step, an AlGaN mesa of 35 × 35 μm was isolated by AlGaN etching. The top cantilever outline was defined by etching down GaN using Cl_2_-based inductively coupled plasma (ICP) etch process in such a way that the AlGaN mesa was at the base of the cantilever. The HFET was then fabricated on the mesa with the source and drain ohmic contacts formed on its two sides by e-beam deposition of Ti (20 nm)/Al (100 nm)/Ti (45 nm)/Au (55 nm) metal stack, followed by a rapid thermal processing (RTP) at 800 °C in the presence of N_2_ for 60 s. The Schottky contact for gate terminal was formed in between the source and drain contacts by depositing metal layers of Ni (25 nm)/Au (375 nm). Next, Ti (20 nm)/Au (150 nm) layer was deposited to form the top bonding pads. Finally, the Si substrate was etched from underneath following the Bosch process, to release the cantilevers. Figure 1b shows the SEM image of a fully fabricated microcantilever, clearly showing the HFET embedded at the base along with the source, gate, and drain contacts. Inset of Figure 1b shows a magnified picture of the AlGaN mesa with a clear image of the HFET. The fabricated devices were then wire-bonded onto a chip carrier for characterization and gas-sensing experiments. 

**Figure 2:**
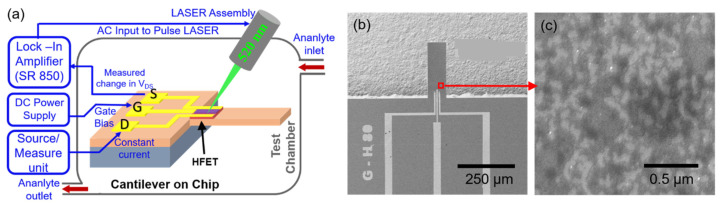
(**a**) Schematic of the experimental setup showing the HFET biasing scheme, with the microcantilever sensor placed inside a test chamber. A 520-nm pulsed laser was shone on the HFET for photoacoustic excitation. (**b**) SEM image of a 1-nm Pd-coated device. (**c**) A magnified SEM image near the HFET of the Pd-coated device (shown with red square) reveals a non-uniform pattern of the deposited 1-nm Pd.

The schematic of the gas-sensing setup is presented in Figure 2, which shows the sensor device assembly enclosed in a test chamber to control analyte flow. Photoacoustic (PA) excitation of the cantilever was realized using a pulsed laser directed on the HFET at the cantilever base. The AlGaN/GaN-based HFET acts as a highly sensitive deflection transducer and converts the cantilever deflection into an electrical signal, details of which have been discussed elsewhere [38,42]. A lock-in amplifier was used to provide a variable frequency 1.1 V rms signal for laser modulation and to record the change in drain-source voltage (ΔV_DS_) from the HFET as a function of frequency. The maximum value of ΔV_DS_ corresponds to the maximum deflection of the cantilever and occurs at its resonance frequency (f_R_). The value of ΔV_DS_ at f_R_ is the resonance amplitude and indicative of the device sensitivity. To measure ΔV_DS_, a constant drain current of 100 μA was maintained by a source measure unit (SMU) and a gate bias (V_G_) was applied at the gate terminal from a DC power supply. The device sensitivity can be tuned using optimized biasing conditions [39]. In addition, plasmonic effects in metal nanoparticles (NPs) can be used to enhance optical absorption and further amplify the resonance amplitude, which in turn, enhances the sensitivity of the device [46]. The deposited metal NPs can also act as a functionalization layer to enable analyte detection [50]. In this work, we used Pt and Pd metal nanoparticles to perform the detection of H_2_ and NH_3_ gases. 1 nm each of Pt and Pd NPs was deposited on the cantilevers using e-beam evaporation utilizing a shadow mask to obtain plasmonic signal enhancement as well as perform selective detection of the analyte gases. High purity Pt (99.99%) and Pd (99.95%) pallets bought from Kurt J. Lesker were used as the targets for e-beam deposition, which was carried out at 3 µTorr and 25 °C using the CCS CA-40 e-beam evaporator. Figure 2b shows am SEM image of a cantilever that was deposited with 1 nm Pd. A higher magnification SEM image (Figure 2c) near the HFET of the device shows the discontinuous nature of the deposited Pd nanostructures. 

## 3. Results and Discussions

**Figure 3:**
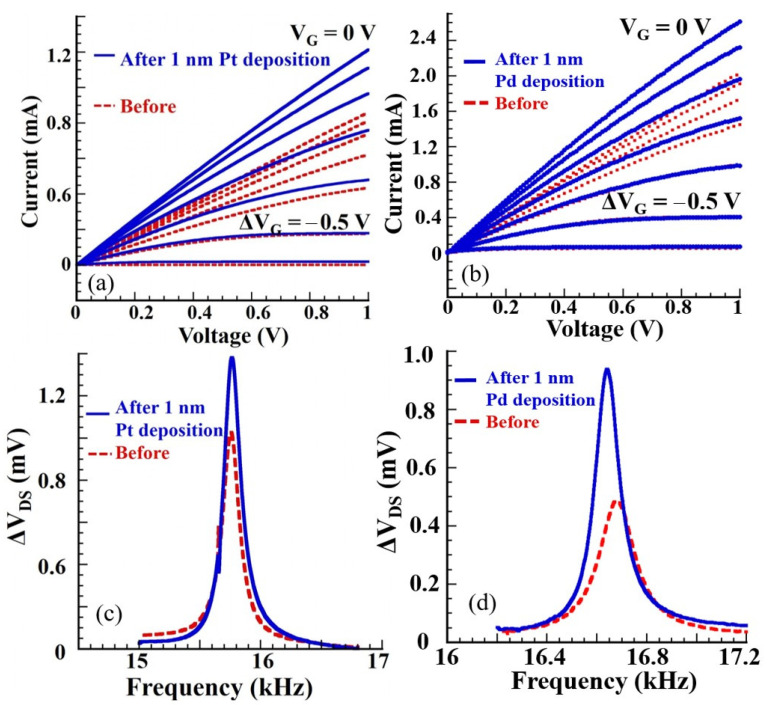
I-V curves of HFET integrated at the cantilever base before and after (**a**) 1-nm Pt deposition and (**b**) 1-nm Pd deposition, showing perfect gate modulation. The resonance characteristics of the device before and after (**c**) 1-nm Pt deposition and (**b**) 1-nm Pd deposition, showing 1.4 and 2 times amplification in the resonance amplitudes, respectively, due to the plasmonic absorption. A red shift of 43 Hz is clearly observed in the resonance frequency in (**d**) due to mass loading of the cantilever following Pd metal deposition.

Figure 3a,b shows the current voltage (I-V) characteristics of the HFET before and after 1-nm Pt and 1-nm Pd deposition, respectively. Both sets of I-V curves show excellent drain current modulation and complete device shutdown at higher (more negative) V_G_, clearly preserving the HFET characteristics even after metal functionalization layer deposition. In fact, an improvement in drain-source saturation current and a reduction in knee voltage was especially noticeable at higher V_G_, which was most likely caused by surface stabilization and a reduction in surface traps as a result of thin metal deposition [51,52,53]. Figure 3c,d shows the resonance responses before and after the 1-nm Pt and Pd deposition, showing enhancement factors of 1.4 and 2, respectively. The laser used for the PA experiments was a 520-nm pulsed module, at which both Pt and Pd NPs were expected to exhibit significant plasmonic absorption [54,55]. Higher enhancement factor for Pd for the same thickness as Pt agreed with the higher plasmonic absorption of Pd NPs at 520 nm [54,55]. Figure 3d also portrays the blue shift in resonance frequency by 43 Hz due to mass loading from the deposited Pd NPs. The same effect was not noticeable in the Pt-deposited device, likely due to more significant surface stress-related changes countering the mass loading effects [30,31]. For gas-sensing experiments the devices were oscillated at their resonance frequency to harness maximum sensitivity arising from quality factor enhancement [29]. 

**Figure 4:**
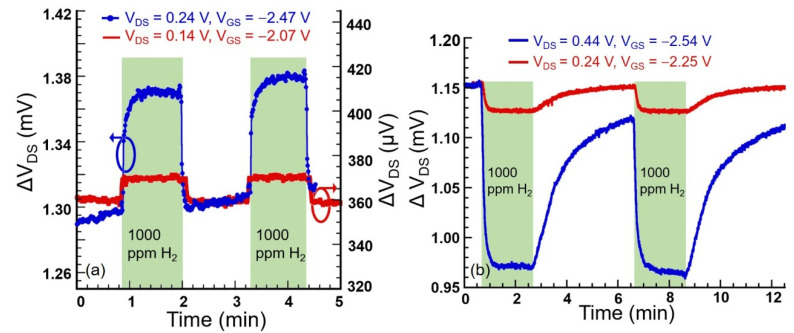
(**a**) Sensor responses to 1000 ppm H_2_ at different biasing conditions (V_DS_ = 0.14, V_GS_ = −2.07 V and V_DS_ = 0.24 V, V_GS_ = −2.47 V) of 1-nm Pt-coated device. Higher change in signal (5-fold enhancement) was recorded for more negative gate bias. SNR value also increased 2 fold, from 25.2 to 51. (**b**) Responses at two different biasing conditions (V_DS_ = 0.24, V_GS_ = −2.25 V and V_DS_ = 0.24 V, V_GS_ = −2.54 V) when exposed to 1000 ppm of H_2_ for 1-nm Pd-coated device. The response magnitude increased from 27 µV to 180 µV, while SNR increased from 30.5 to 59.9 at higher bias.

Figure 4a shows the response of 1-nm Pt-coated device upon exposure to 1000 ppm H_2_. Response amplitude was found to depend on the biasing condition of the device as the sensitivity of the AlGaN/GaN heterostructure increased with higher V_G_ [39], which was reflected in higher resonance amplitude at higher (more negative) gate biases. As seen in Figure 4a, for the same concentration of H_2_ (1000 ppm) and flow rate (250 sccm, controlled by mass flow controllers), when the device was biased at V_GS_ = −2.07 V (and V_DS_ = 0.14 V), the response amplitude was found to be 15 µV (signal-to-noise ratio (SNR) = 25.2), which increased 5 folds (SNR = 51) when the device was biased at V_GS_ = −2.47 V (and V_DS_ = 0.24 V). Similar trend was also exhibited by the Pd-coated device, as can be seen from Figure 4b, where the response amplitude increased dramatically from 27 µV (SNR = 30.5) to 180 µV (SNR = 59.9) when the biasing condition was changed from V_GS_ = −2.25 V (and V_DS_ = 0.24 V) to V_GS_ = −2.54 V (V_DS_ = 0.44 V), for the same concentration (1000 ppm) and flow rate (250 sccm) of H_2_. 

The usage of Pt and Pd NPs for H_2_ detection is quite common as H_2_ atoms can easily and selectively adsorb in the Pt and Pd lattice by moving into the interstitial spaces and forming metal hydrides [9,10]. The solubility of H_2_ is further improved in nanoscale because of increased surface area [56]. Moreover, the adsorbed H_2_ changes the shape, size, and, therefore, the aspect ratio as well as the dielectric environment of the Pd NPs [8], modifying their plasmonic properties. This causes a change in the plasmonic absorption spectra, with a resonance peak shift as well as a peak amplitude change, which makes the usage of plasmonic NPs an even more effective way of detecting H_2_ [8]. For Pt NPs a definite adsorption model has not been established yet, but there is evidence that the dissociative surface adsorption of H_2_ on Pt surface [56,57,58] facilitates the change in the electronic configuration as well as in the dielectric environment of the Pt NPs, which can be instrumental in altering the plasmonic excitation in the same. These modifications in their absorption spectra changes the strength of the PA wave generated due to the plasmonic excitation of these particles, which can then be measured by the change in the HFET output in terms of change in ∆V_DS_. We also noted the opposite nature of the change in signal upon H_2_ exposure (increase in the magnitude of the Pt-coated device and a reduction for the Pd-coated device), which likely originated from the opposite phase of the HFET output signal recorded on these devices.

**Figure 5:**
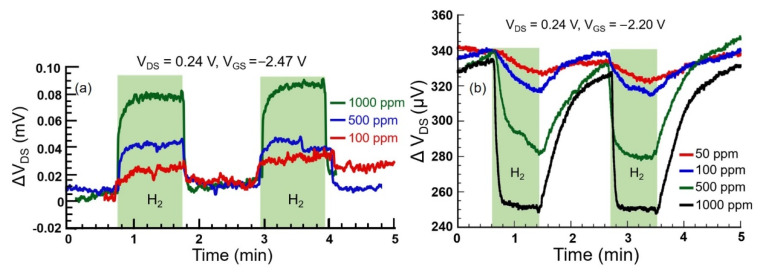
(**a**) Responses to three different concentrations of H_2_ for 1-nm Pt-coated device, showing response magnitudes of 75 and 20 µV as the H_2_ concentration changes from 1000 ppm to 100 ppm, with SNR changing from 51 to 19.9 (**b**) Response to three different concentrations of H_2_ of 1-nm Pd-coated device, showing magnitudes of 85 and 13 µV as the H_2_ concentration changes from 1000 ppm to 50 ppm, with SNR changing from 116.4 to 11.8.

To compare the sensing performances of the Pt- and Pd-coated devices, they were biased at the same V_DS_ and exposed to different concentrations of H_2_. Figure 5a shows the responses of the Pt-coated device upon exposure to 1000, 500, and 100 ppm of H_2_ while biased at V_GS_ = −2.47 V and V_DS_ = 0.24 V, and oscillated at resonance frequency of f_R_ = 15.762 kHz. Figure 5b shows the responses for a Pd-coated device at the same drain bias (V_GS_ = −2.20 V and V_DS_ = 0.24 V) and in resonant mode (f_R_ = 15.65 kHz), upon exposure to H_2_ of different concentrations, varying from 1000 ppm to 50 ppm. We observed a much cleaner signal for the Pd-coated device with much higher SNR, which enabled clear identification of 50 ppm H_2_ response, while even 100 ppm H_2_ response was barely detectable over the background noise in the Pt-coated device. For a better comparison between the Pt- and Pd-coated devices, response time, SNR, and limit of detection (LOD), corresponding to various H_2_ concentrations, as extracted from the sensor responses presented in Figure 5, are summarized in Table 1. The response time, which is defined between the 10th and 90th percentile point, i.e., (τ*_10%_*–τ*_90%_*), was recorded to be 9 s in the Pd device for its response to 1000 ppm H_2_. The same for the Pt-coated device was found to be much higher at 13 s. The lowest SNR value of 19.9 was recorded for Pt-coated device at 100 ppm H_2_, showing that the LOD of the Pt-coated device for the given biasing condition was ~15 ppm, using the LOD definition as the lowest concentration that can be measured with 3 or higher SNR [59]. 

However, for the Pd-coated device, the lowest SNR corresponding to 50 ppm H_2_ was recorded to be 11.8, indicating that the LOD was ~12 ppm. The H_2_ adsorption mechanism in the bulk as well as in nanoscale Pd and Pt has been studied extensively by many researchers. While the H_2_ diffusion coefficients on Pt and Pd are almost equal, the H_2_ solubility in Pd is about three orders of magnitude higher than that in Pt [6,60], which can be attributed to the superior performance of the Pd-functionalized devices. As we saw in Figure 4, the SNR performance of the Pd-coated devices can be improved with optimization of the biasing conditions, which indicates that the LOD of the Pd device can be improved with further optimization of the biasing conditions. Nonetheless, the LOD obtained for our device using plasmonically enhanced photoacoustic detection technique is very comparable to the LOD exhibited by widely used surface plasmon resonance (SPR)-based techniques (lowest LOD was ~10 ppm), the detection methods of which are much more cumbersome and bulky [61,62]. The performance of the Pd-coated devices was also tested when the analyte was diluted with air (with 60% relative humidity) and compared with the same when the analyte was diluted with N_2_. While the response magnitude remained comparable, the transient time was found to improve with air dilution. The corresponding data are presented in the Supplementary Figure S1.

**Figure 6:**
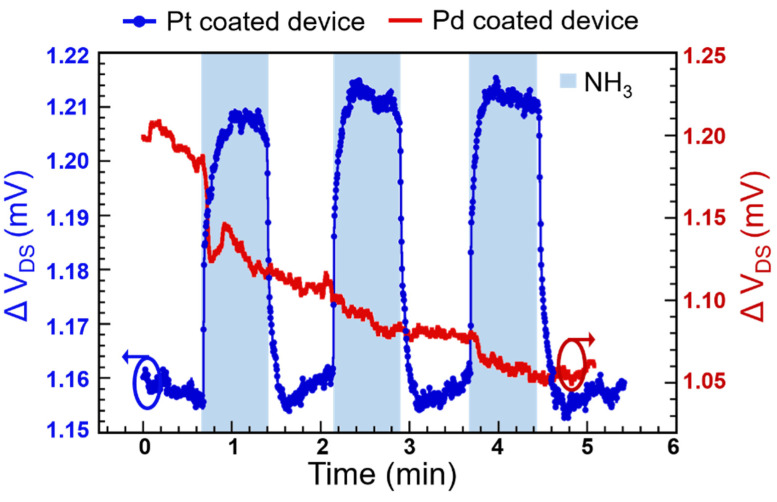
Responses of the Pt- and Pd-coated sensor devices upon exposure to 500 ppm NH_3_ over three test cycles. While the Pt-coated device shows a repeatable response of 50 µV with an SNR of 85, the Pd-coated device does not recover after the initial drop in signal or show further response to subsequent cycles of NH_3_ exposure.

NH_3_ is also adsorbed in several metal films, including Pt and Pd, although the individual mechanisms of adsorption are complicated and may vary widely [11,12,13,63]. We investigated the detection performance of the Pt and Pd NP-coated devices with respect to NH_3_ sensing. The results are shown in Figure 6 for three cycles of 500 ppm NH_3_ flow. We found that while the Pt-coated devices exhibited repeatable and sensitive response to 500 ppm NH_3_, the Pd-coated devices exhibited no significant response after the initial drop (60 µV) in HFET output upon the first exposure to NH_3_ and did not recover or respond to the subsequent NH_3_ exposures. The Pt-coated device, when biased at V_DS_ = 0.14 V and V_GS_ = −2.07 V, exhibited a response amplitude of 50 µV with an SNR of 85 (second response cycle in Figure 6 was used), and clear response and recovery transients for all the exposure cycles to 500 ppm NH_3_. From this response and the SNR value, the LOD (with SNR of 3) was found to be ~17 ppm. The response time from the second cycle was also found to be 6 s based on the definition of response time discussed earlier. The LOD and response time for NH_3_ detection was superior to those of the SPR-based techniques, where the best LODs and response times reported were ~10 ppm and a few tenths of a second, respectively [64,65,66]. We also note that the lack of response from the Pd NP-functionalized sensor device pointed out the unique possibility of selective detection of H_2_ over NH_3_, which is important for applications where these two reducing gases are present simultaneously [67,68]. The lack of specific changes might be related to ready dissociation of NH_3_ following its adsorption in Pd [11]. 

## 4. Conclusions

In conclusion, we successfully demonstrated a unique and sensitive detection of H_2_ using Pt and Pd plasmonic NPs as functionalization layers in GaN microcantilevers using photoacoustic excitation at 520 nm. A comparison of H_2_-sensing performance between Pt and Pd NPs-coated devices indicated superior sensing performance of Pd-coated devices in terms of detection limit and response time. Strong dependence on the biasing conditions was observed, indicating the possibility of improving the sensing performance with further bias optimization. Excellent sensing performance of the Pt-functionalized devices for NH_3_ detection was observed, which exhibited a LOD of ~17 ppm and fast response time, while the Pd-functionalized device did not produce any significant response, indicating the possibility of selective detection of H_2_ over NH_3_ using the latter. 

## Figures and Tables

**Table 1:** Summary of Pd- and Pt-functionalized sensor data showing comparison of their sensing performances.

NP	H_2_ Concentration	Response Time	Signal-to-Noise Ratio (SNR)	Limit of Detection (LOD)
Pt	1000 ppm	13 s	51	~15 ppm
	500 ppm	7 s	33.9	
	100 ppm	12 s	19.9	
Pd	1000 ppm	9 s	116.4	~12 ppm
	500 ppm	13 s	104.1	
	100 ppm	20 s	36.7	
	50 ppm	26 s	11.8	

## Supplementary Materials

The following are available online at https://www.mdpi.com/2072-666X/11/7/680/s1, Figure S1: Response of the Pd coated device to 500 ppm H_2_ diluted in UHP N_2_ and in ambient air with 60% relative humidity. Clear improvement in response is observed for air dilution.

## References

[1] Cashdollar K.L., Zlochower I.A., Green G.M., Thomas R.A., Hertzberg M. (2000). Flammability of methane, propane, and hydrogen gases. J. Loss Prev. Process. Ind..

[2] Licht S., Cui B., Wang B., Li F.-F., Lau J., Liu S. (2014). Ammonia synthesis by N2 and steam electrolysis in molten hydroxide suspensions of nanoscale Fe2O3. Science.

[3] Barbir F. (2005). PEM electrolysis for production of hydrogen from renewable energy sources. Sol. Energy.

[4] Chen P., Wu X., Lin J., Tan K.L. (1999). High H2 Uptake by Alkali-Doped Carbon Nanotubes Under Ambient Pressure and Moderate Temperatures. Science.

[5] Zhang D., Liu Z., Li C., Tang T., Liu X., Han S., Lei B., Zhou C. (2004). Detection of NO2down to ppb Levels Using Individual and Multiple In2O3Nanowire Devices. Nano Lett..

[6] Kim C.K., Lee J., Choi S., Noh I., Kim H., Cho N., Hong C., Jang G. (2001). Pd- and Pt-SiC Schottky diodes for detection of H2 and CH4 at high temperature. Sens. Actuators B Chem..

[7] Cheng C.-C., Tsai Y.-Y., Lin K.-W., Chen H.-I., Liu W.-C. (2005). Hydrogen sensing properties of a Pt-oxide-Al0.24Ga0.76As high-electron-mobility transistor. Appl. Phys. Lett..

[8] Wadell C., Syrenova S., Langhammer C. (2014). Plasmonic Hydrogen Sensing with Nanostructured Metal Hydrides. ACS Nano.

[9] Isobe Y., Yamauchi M., Ikeda R., Kitagawa H. (2003). A study on hydrogen adsorption of polymer protected Pt nanoparticles. Synth. Met..

[10] Shin W., Imai K., Izu N., Murayama N. (2001). Thermoelectric Thick-Film Hydrogen Gas Sensor Operating at Room Temperature. Jpn. J. Appl. Phys..

[11] Stolbov S., Rahman T.S. (2005). First Principles Study of Adsorption, Diffusion and Dissociation of NH_3 on Ni and Pd Surfaces. arXiv.

[12] Offermans W., Jansen A., Van Santen R., Novell-Leruth G., Ricart J., Perez-Ramirez J. (2007). Ammonia Dissociation on Pt {100}, Pt {111}, and Pt {211}: A Comparative Density Functional Theory Study. J. Phys. Chem. C.

[13] Gohndrone J.M. (1989). Ammonia adsorption and decomposition on several faces of platinum. J. Vac. Sci. Technol. A.

[14] Lupan O., Postica V., Labat F., Ciofini I., Pauporté T., Adelung R. (2018). Ultra-sensitive and selective hydrogen nanosensor with fast response at room temperature based on a single Pd/ZnO nanowire. Sens. Actuators B Chem..

[15] Buttner W., Post M.B., Burgess R., Rivkin C. (2011). An overview of hydrogen safety sensors and requirements. Int. J. Hydrogen Energy.

[16] Wu J., Fedder G.K., Carley L.R. (2004). A Low-Noise Low-Offset Capacitive Sensing Amplifier for a 50-/Spl Mu/G//Spl Radic/Hz Monolithic CMOS MEMS Accelerometer. IEEE J. Solid-State Circuits.

[17] Hanay M.S., Kelber S., Naik A.K., Chi D., Hentz S., Bullard E.C., Colinet E., Duraffourg L., Roukes M.L. (2012). Single-protein nanomechanical mass spectrometry in real time. Nat. Nanotechnol..

[18] Abeysinghe D., Dasgupta S., Boyd J., Jackson H. (2001). A novel MEMS pressure sensor fabricated on an optical fiber. IEEE Photon- Technol. Lett..

[19] Gajula D., Jahangir I., Koley G. (2018). High Temperature AlGaN/GaN Membrane Based Pressure Sensors. Micromachines.

[20] Jha C.M., Bahl G., Melamud R., Chandorkar S.A., Hopcroft M.A., Kim B., Agarwal M., Salvia J., Mehta H., Kenny T.W. Cmos-Compatible Dual-Resonator MEMS Temperature Sensor with Milli-Degree Accuracy. Proceedings of the TRANSDUCERS 2007—2007 International Solid-State Sensors, Actuators and Microsystems Conference.

[21] Azevedo R.G., Jones D.G., Jog A.V., Jamshidi B., Myers D.R., Chen L., Fu X.-A., Mehregany M., Wijesundara M.B.J., Pisano A.P. (2007). A SiC MEMS Resonant Strain Sensor for Harsh Environment Applications. IEEE Sens. J..

[22] Augustyniak I., Dziuban J., Knapkiewicz P., Matusiak M., Olszacki M., Pons P. MEMS high-doses radiation sensor. Proceedings of the 2013 Transducers & Eurosensors XXVII: The 17th International Conference on Solid-State Sensors, Actuators and Microsystems (TRANSDUCERS & EUROSENSORS XXVII).

[23] Voiculescu I., Nordin A.N. (2012). Acoustic wave based MEMS devices for biosensing applications. Biosens. Bioelectron..

[24] Nomani W., Kersey D., James J., Diwan D., Vogt T., Webb R.A., Koley G. (2011). Highly sensitive and multidimensional detection of NO2 using In2O3 thin films. Sens. Actuators B Chem..

[25] Singh A., Uddin A., Sudarshan T., Koley G. (2013). Tunable Reverse-Biased Graphene/Silicon Heterojunction Schottky Diode Sensor. Small.

[26] Singh A.K., Uddin M.A., Tolson J.T., Maire-Afeli H., Sbrockey N., Tompa G.S., Spencer M.G., Vogt T., Sudarshan T.S., Koley G. (2013). Electrically tunable molecular doping of graphene. Appl. Phys. Lett..

[27] Uddin A., Singh A., Sudarshan T.S., Koley G. (2014). Functionalized graphene/silicon chemi-diode H2 sensor with tunable sensitivity. Nanotechnology.

[28] Craighead H.G. (2000). Nanoelectromechanical Systems. Science.

[29] Tamayo J. (2005). Study of the noise of micromechanical oscillators under quality factor enhancement via driving force control. J. Appl. Phys..

[30] Pinnaduwage L.A., Boiadjiev V., Hawk J.E., Thundat T. (2003). Sensitive detection of plastic explosives with self-assembled monolayer-coated microcantilevers. Appl. Phys. Lett..

[31] Huang X.M.H., Manolidis M., Jun S.C., Hone J. (2005). Nanomechanical hydrogen sensing. Appl. Phys. Lett..

[32] Koley G., Qazi M., Lakshmanan L., Thundat T. (2007). Gas sensing using electrostatic force potentiometry. Appl. Phys. Lett..

[33] Fan R., Zolper J.C. (2003). Wide Energy Bandgap Electronic Devices.

[34] Ambacher O., Smart J., Shealy J., Weimann N.G., Chu K., Murphy M., Schaff W.J., Eastman L.F., Dimitrov R., Wittmer L. (1999). Two-dimensional electron gases induced by spontaneous and piezoelectric polarization charges in N- and Ga-face AlGaN/GaN heterostructures. J. Appl. Phys..

[35] Zhou Q., Wong K.-Y., Chen W., Chen K.J. (2010). Wide-Dynamic-Range Zero-Bias Microwave Detector Using AlGaN/GaN Heterojunction Field-Effect Diode. IEEE Microw. Wirel. Components Lett..

[36] Villanueva L.G., Plaza J.A., Montserrat J.M., Perez F., Bausells J. (2008). Crystalline silicon cantilevers for piezoresistive detection of biomolecular forces. Microelectron. Eng..

[37] Thaysen J., Boisen A., Hansen O., Bouwstra S. (2000). Atomic force microscopy probe with piezoresistive read-out and a highly symmetrical Wheatstone bridge arrangement. Sens. Actuators A Phys..

[38] Qazi M., DeRoller N., Talukdar A., Koley G. (2011). III-V Nitride based piezoresistive microcantilever for sensing applications. Appl. Phys. Lett..

[39] Talukdar A., Koley G. (2014). Impact of Biasing Conditions on Displacement Transduction by III-Nitride Microcantilevers. IEEE Electron Device Lett..

[40] Bayram F., Khan D., Li H., Hossain M., Koley G. (2018). Piezotransistive GaN microcantilevers based surface work function measurements. Jpn. J. Appl. Phys..

[41] Bayram F., Gajula D., Khan D., Gorman S., Koley G. (2019). Nonlinearity in piezotransistive GaN microcantilevers. J. Micromech. Microeng..

[42] Talukdar A., Khan M.F., Lee N., Kim S., Thundat T., Koley G. (2015). Piezotransistive transduction of femtoscale displacement for photoacoustic spectroscopy. Nat. Commun..

[43] Catchpole K.R., Polman A. (2008). Plasmonic solar cells. Opt. Express.

[44] Reineck P., Lee G.P., Brick D., Karg M., Mulvaney P., Bach U. (2012). A Solid?State Plasmonic Solar Cell via Metal Nanoparticle Self?Assembly. Adv. Mater..

[45] Genevet P., Lin J., Kats M.A., Capasso F. (2012). Holographic detection of the orbital angular momentum of light with plasmonic photodiodes. Nat. Commun..

[46] Khan D., Bayram F., Gajula D., Talukdar A., Li H., Koley G. (2017). Plasmonic amplification of photoacoustic waves detected using piezotransistive GaN microcantilevers. Appl. Phys. Lett..

[47] Rashid T.-R., Phan D.-T., Chung G.-S. (2013). A flexible hydrogen sensor based on Pd nanoparticles decorated ZnO nanorods grown on polyimide tape. Sens. Actuators B Chem..

[48] Tobiska P., Hugon O., Trouillet A., Gagnaire H. (2001). An integrated optic hydrogen sensor based on SPR on palladium. Sensors Actuators B: Chem..

[49] Mishra S.K., Kumari D., Gupta B.D. (2012). Surface plasmon resonance based fiber optic ammonia gas sensor using ITO and polyaniline. Sens. Actuators B Chem..

[50] Khan D., Gajula D., Bayram F., Koley G. Plasmonic Absorption Enabled Analyte Detection Using Piezotransistive Microcantilevers. Proceedings of the 2018 IEEE 13th Nanotechnology Materials and Devices Conference (NMDC).

[51] Kim H., Thompson R., Tilak V., Prunty T., Shealy J., Eastman L.F. (2003). Effects of SiN passivation and high-electric field on AlGaN-GaN HFET degradation. IEEE Electron Device Lett..

[52] Kordos P., Kudela P., Gregušová D., Donoval D. (2006). The effect of passivation on the performance of AlGaN/GaN heterostructure field-effect transistors. Semicond. Sci. Technol..

[53] Green B., Chu K., Chumbes E., Smart J., Shealy J., Eastman L.F. (2000). The effect of surface passivation on the microwave characteristics of undoped AlGaN/GaN HEMTs. IEEE Electron Device Lett..

[54] Ghosh S., Nitnavare R., Dewle A., Tomar G.B., Chippalkatti R., More P., Kitture R., Kale S., Bellare J., Chopade B.A. (2015). Novel platinum–palladium bimetallic nanoparticles synthesized by Dioscorea bulbifera: Anticancer and antioxidant activities. Int. J. Nanomed..

[55] Huang X., Tang S., Mu X., Dai Y., Chen G., Zhou Z.-Y., Ruan F., Yang Z., Zheng N. (2010). Freestanding palladium nanosheets with plasmonic and catalytic properties. Nat. Nanotechnol..

[56] Yamauchi M., Kobayashi H., Kitagawa H. (2009). Hydrogen Storage Mediated by Pd and Pt Nanoparticles. ChemPhysChem.

[57] Kemppainen E., Bodin A., Sebok B., Pedersen T., Seger B., Mei B., Bae D., Vesborg P.C.K., Halme J., Hansen O. (2015). Scalability and feasibility of photoelectrochemical H2evolution: The ultimate limit of Pt nanoparticle as an HER catalyst. Energy Environ. Sci..

[58] Arboleda N.B., Kasai H., Diño W.A., Nakanishi H. (2007). Potential Energy of H2Dissociation and Adsorption on Pt(111) Surface: First-Principles Calculation. Jpn. J. Appl. Phys..

[59] Kim N.-H., Choi S.-J., Yang D.-J., Bae J., Park J., Kim I.-D. (2014). Highly sensitive and selective hydrogen sulfide and toluene sensors using Pd functionalized WO3 nanofibers for potential diagnosis of halitosis and lung cancer. Sens. Actuators B Chem..

[60] Lechuga L.M., Calle A., Golmayo D., Briones F. (1992). Different catalytic metals (Pt, Pd and Ir) for GaAs Schottky barrier sensors. Sens. Actuators B Chem..

[61] Watkins W.L., Borensztein Y. (2018). Ultrasensitive and fast single wavelength plasmonic hydrogen sensing with anisotropic nanostructured Pd films. Sens. Actuators B Chem..

[62] Ndaya C.C., Javahiraly N., Brioude A. (2019). Recent Advances in Palladium Nanoparticles-Based Hydrogen Sensors for Leak Detection. Sensors.

[63] Gland J.L., Kollin E.B. (1981). Ammonia adsorption on the Pt(111) AND Pt(S)-6(111) × (111) surfaces. Surf. Sci..

[64] Mishra S.K., Tripathi S.N., Choudhary V., Gupta B.D. (2014). SPR based fibre optic ammonia gas sensor utilizing nanocomposite film of PMMA/reduced graphene oxide prepared by in situ polymerization. Sens. Actuators B Chem..

[65] Aarya S., Kumar Y., Chahota R.K. (2019). Recent Advances in Materials, Parameters, Performance and Technology in Ammonia Sensors: A Review. J. Inorg. Organomet. Polym. Mater..

[66] Bhatia P., Gupta B.D. (2012). Surface Plasmon Resonance Based Fiber Optic Ammonia Sensor Utilizing Bromocresol Purple. Plasmonics.

[67] Modak J. (2002). Haber process for ammonia synthesis. Resonance.

[68] Choudhary T., Sivadinarayana C., Goodman D. (2001). Catalytic ammonia decomposition: COx-free hydrogen production for fuel cell applications. Catal. Lett..

