# Supplementary material for: Photoacoustic Detection of H2 and NH3 Using Plasmonic Signal Enhancement in GaN Microcantilevers
Source: Micromachines (Basel). 2020 Jul 13;11(7):680. doi: 10.3390/mi11070680 (PMC7407488; doi:10.3390/mi11070680)
Supplement: Supplementary file 1 [file micromachines-11-00680-s001.pdf]

micromachines

Supplementary Materials: Photoacoustic Detection of H_2 and NH_3 Using Plasmonic Signal Enhancement in GaN Microcantilevers

Digangana Khan [1,*], Hongmei Li [1], Ferhat Bayram [1], Durga Gajula [2] and Goutam Koley [1]

1. Holcombe Department of Electrical and Computer Engineering, Clemson University, Clemson, SC 29634, USA; hongmel@g.clemson.edu (H.L.); fbayram@g.clemson.edu (F.B.); gkoley@clemson.edu (G.K.)
2. School of Electrical and Computer Engineering, Georgia Institute of Technology, Atlanta, GA 30332, USA; gdraophy@gmail.com

* Correspondence: digangk@g.clemson.edu; Tel.: +1-(864)-656-4698

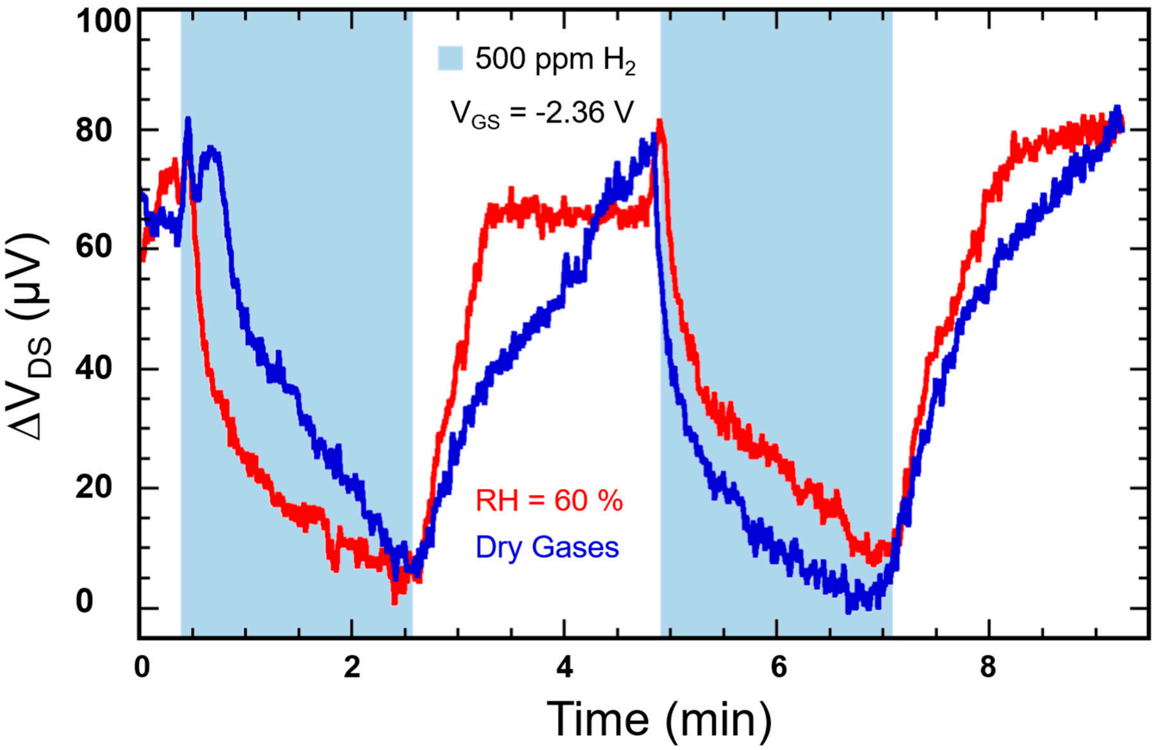

Figure S1. Response of the Pd coated device to 500 ppm H_2 diluted in UHP N_2 and in ambient air with 60% relative humidity. Clear improvement in response is observed for air dilution.
